# Sulfation of sialic acid is ubiquitous and essential for vertebrate development

**DOI:** 10.1038/s41598-022-15143-4

**Published:** 2022-07-21

**Authors:** Nursah Ertunc, Thanyaluck Phitak, Di Wu, Hiroshi Fujita, Masaya Hane, Chihiro Sato, Ken Kitajima

**Affiliations:** 1 Bioscience and Biotechnology Center, and Graduate School of Bioagricultural Sciences, Nagoya University, Nagoya, 464-8601 Japan; 2 Institute for Glyco-Core Research (iGCORE), Nagoya University, Nagoya, 464-8601 Japan; 3 Present Address: Molecular Cell Biology, Faculty of Medical Technology, Graduate School of Health Sciences, Fujita Health University, 1-98 Dengakugakubo, Kutsukake, Toyoake, Aichi 470-1192 Japan; 4 Present Address: Biochemistry Department, Faculty of Medicine, Chiangmai University, Chiangmai, 50200 Thailand

**Keywords:** Glycobiology, Transferases

## Abstract

Glycosylation of proteins and lipids occurs in vertebrates, usually terminating with sialylation, which regulates the physicochemical and biological properties of these glycoconjugates. Although less commonly known, sialic acid residues also undergo various modifications, such as acetylation, methylation, and sulfation. However, except for acetylation, the enzymes or functions of the other modification processes are unknown. To the best of our knowledge, this study is the first to demonstrate the ubiquitous occurrence of sulfated sialic acids and two genes encoding the sialate: *O*-sulfotransferases 1 and 2 in vertebrates. These two enzymes showed about 50% amino acid sequence identity, and appeared to be complementary to each other in acceptor substrate preferences. Gene targeting experiments showed that the deficiency of these genes was lethal for medaka fish during young fry development and accompanied by different phenotypes. Thus, the sulfation of sialic acids is essential for the vertebrate development.

## Introduction

Sialic acids (Sias) are a group of nine-carbon carboxylated sugars that modify the termini of glycans of proteins and lipids on the cell surface and the extracellular matrix^1–3^. Sias have been demonstrated to play an essential role for embryo survival in vertebrates, because loss of critical enzymes in the Sia metabolic pathway leads to the embryonic death. Mice deficient in the UDP-*N*-acetylglucosamine (GlcNAc) epimerase/*N*-acetylmannosamine (ManNAc) kinase (*GNE*) gene or the CMP-sialic acid synthetase (*CMAS*) gene are lethal during early development^4,5^. Medaka fish with a point-mutated *CMAS* gene are also lethal in young fry^6^. Sias mediate and regulate various cellular recognition and signaling events^1,2^. For example, sialylation of podocalyxin in the mouse kidney is essential for the formation of glomerular filtration^7^. Polysialylation of the neural cell adhesion molecule (NCAM) in the mouse embryonic brain not only negatively regulates the NCAM homophilic binding^8,9^, but also retain neurotrophins, fibroblast growth factor 2, and dopamine to control their signal transductions^10–13^.

One of unique features of Sia absent from other monosaccharides is that Sia displays a marked structural diversity owing to modifications such as acetylation, methylation, and sulfation^1,14^. However, the metabolism and biological significance of the modified forms of Sia is unclear, except for *O*-acetylated Sia (SiaAc)^15^. *O*-Acetylation of Sia occurs in glycans in proteins and lipids of a wide range of organisms from bacteria to vertebrates^14,16^. SiaAc is involved in murine development at the 2-cell stage^17^, regulation of apoptosis^18,19^ and immune recognition^20–24^. SiaAc-containing gangliosides are prominently expressed in various cancer, and can be a target for cancer immunotherapy^25^. The cell surface SiaAcs are also utilized as targets for bacterial and viral infection^15–28^. Thus, a myriad of information on occurrence and functions of SiaAc have been accumulated. On the other hand, a single mammalian sialic acid *O*-acetyltransferase (SOAT), or CASD1 (capsule structural domain containing 1) has been so far identified as a biosynthetic enzyme^29,30^.

**Figure 1:**
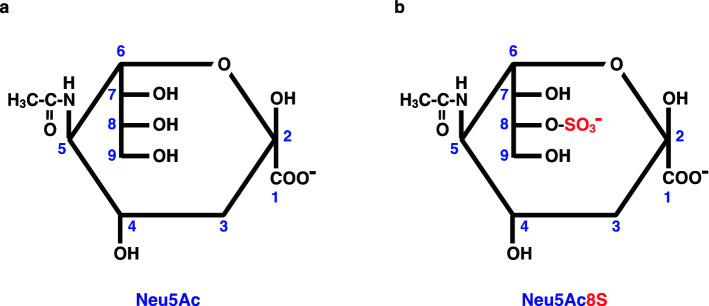
Structure of SiaS. (**a**) *N*-acetylneuraminic acid (Neu5Ac); (**b**) 8-*O*-sulfated *N*-acetylneuraminic acid (Neu5Ac8S).

*O*-Sulfation of Sia has not been studied well, compared with the Sia *O*-acetylation. Sulfated Sia (SiaS; Fig. 1) is identified in glycolipids^31–34^ and glycoproteins^35,36^ of sea urchin gametes, and its importance in sperm-egg interaction^37^ and sperm motility^38,39^ has been demonstrated in sea urchin. In mammals, the occurrence of SiaS is only reported in bovine gastric mucosa^40,41^, human blood^42^, rodent various tissues^43^, and mucin glycoproteins^44^. However, no comprehensive study on the distribution, structural features, biosynthesis, and functional significance of SiaS in other organisms than sea urchin has been performed. Thus, our objective was to obtain the first overview of the biological significance of SiaS in vertebrates.

## Results

### Ubiquitous occurrence of SiaS in mammal

**Figure 2:**
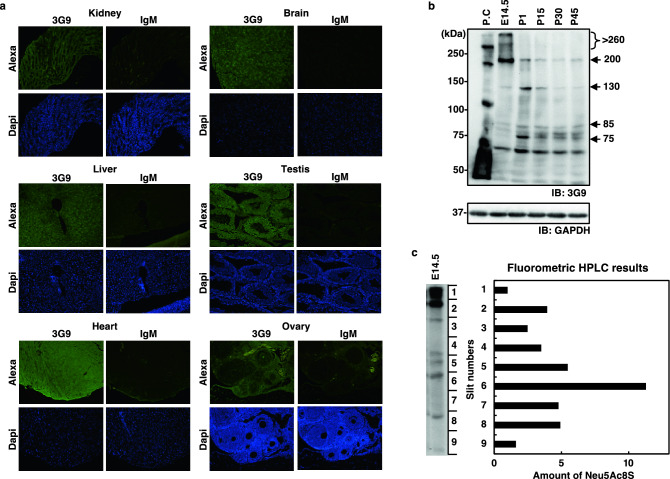
Ubiquitous occurrence of SiaS in mouse. (**a**) Immunohistochemistry of mouse adult tissue sections with the anti-SiaS antibody 3G9. The sections were treated with 3G9 and its isotype control (2G9), followed by the Alexa-488-conjugated anti-mouse IgM treatment to visualize the SiaS epitopes (Alexa, green). Nuclei are stained with DAPI (Dapi, blue); (**b**) Western blotting of the mouse brains with 3G9 and GAPDH (loading control). Lane P.C: sea urchin sperm as positive control; lane E14.5: 14.5 days post-fertilization; lanes P1, P15, P30, and P45: 1, 15, 30, and 45 days after birth. The arrows denote the 3G9-epitope-containing components, and their molecular masses are indicated; (**c**) Chemical detection of SiaS in the mouse brain at E14.5. The brain homogenate was subjected to SDS-PAGE, followed by the transfer to a PVDF membrane. The left edge of the membrane was removed and used for immunostaining with 3G9 (*left panel*). The rest of the membrane was cut into nine pieces according to the molecular size, and analyzed by fluorometric HPLC (*right panel*).

First, we investigated the occurrence and distribution of SiaS in various vertebrate cells and tissues by immunodetection with the 3G9 monoclonal antibody, which specifically recognizes 8-*O*-sulfated *N*-acetylneuraminic acid (Neu5Ac8S)^45,46^. SiaS was detected in all the examined tissue sections from mice and humans (Fig. 2a, Supp_FigS1), including the kidney, liver, brain, heart, testis, and ovary tissues. Developmental expression of SiaS in the brain was also investigated by western blotting using 3G9 (Fig. 2b). The SiaS epitope was detected at a > 250 kDa smear in fetal brain tissue acquired at 14.5 days post-fertilization (E14.5), while < 100 kDa components were increased in neonates (Fig. 2b). SiaS was also chemically detected in the embryonic brain at E14.5 by quantifying the amount of Neu5Ac8S on every slit of the blotted membrane after sodium dodecyl sulfate–polyacrylamide gel electrophoresis (SDS-PAGE) and fluorometric high-performance liquid chromatography (HPLC) analysis (Fig. 2c). The amount of Neu5Ac8S was prominent in slit 6 at approximately 65 kDa, although it was also detected in all other slits. The findings indicated that SiaS is expressed in various organs in mice and humans, and in a developmental stage-dependent manner in the mouse brain. The findings further indicated that the ubiquity of SiaS in mammals is much more frequent than has been recognized.

### cDNA cloning of the sialate: *O*-sulfotransferase (SulT-Sia)

**Figure 3:**
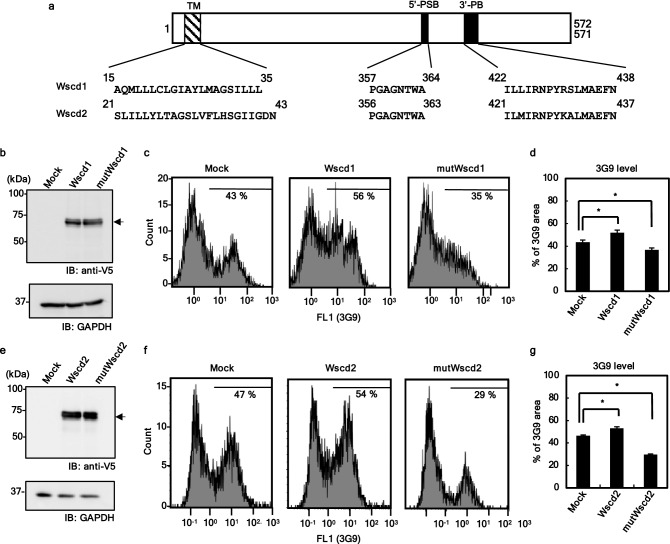
Identification of *Wscd1* and *Wscd2* as SulT-Sia genes. (**a**) Schematic structure of Wscd1 and Wscd2. Both proteins share the two PAPS-binding motifs, 5′-PSB and 3′-PB, and a single membrane-spanning region, TM. Their amino acid sequences are shown. The schematic structure of the alanine mutants, mutWscd1 and mutWscd2, are presented in Supp_FigS4. (**b**, **e**) Western blotting of *Mock*-, *Wscd1*- *Wscd2*-, mut*Wscd1*-, and mut*Wscd2*-transfected CHO cells using antibodies against V5 and GAPDH (loading control). In (**b**), Lanes 1–3: *Mock*-, *Wscd1*-, and *mutWscd1*-transfected cells, respectively. In (**e**), Lanes 1–3: *Mock*-, *Wscd2*-, and *mutWscd2*-transfected cells, respectively. Wscd1, Wscd2, mutWscd1, and mutWscd2 were detected at 71 kDa (arrow). (**c**, **f**) Flow cytometry analysis (FCA) of 3G9 epitope expression on the transfected cells shown in (**b**) and (**e**), respectively. The samples include Mock, Wscd1-expressing, and mutWscd1-expressing cells in (**c**); Mock, Wscd2-expressing, and mutWscd2-expressing cells in (**f**). (**d**, **g**) The % proportion of 3G9-positive cell population of the histograms in (**c**) and (**f**), respectively. All the experiments were performed in triplicate. The error bars indicate the standard deviations; *p < 0.05 (Student t-test, n = 3).

We next sought to clone the gene for an *O*-sulfotransferase enzyme responsible for transferring the sulfonyl group to the hydroxy group of Sia (SulT-Sia). Based on the two conserved motifs of the 3′-phosphoadenosine-5′-phosphosulfate (PAPS)-binding domain among sulfotransferases, 61 genes that were already annotated as sulfotransferases in mice were selected (Supp_DataS1). Although the acceptor substrates for most had already been predicted, two genes appeared to code for sulfotransferases of unknown acceptor substrate specificity: *Wscd1* (wall integrity and stress response component [WSC] domain containing 1; NCBI Gene ID: 216881) and *Wscd2* (WSC domain containing 2; NCBI Gene ID: 320916). *Wscd1* and *Wscd2* full-length cDNA was cloned by RT-PCR using total RNA from the E14.5 mouse embryonic brain (Supp_FigS2a). The nucleotide sequences of *Wscd1* and *Wscd2* contained open reading frames of 1719 bp and 1716 bp, respectively, encoding 572 and 571 amino acid residues (Supp_FigS2b, Fig. 3a). The mouse Wscd1 (mWscd1) and mWscd2 showed 49% identity and shared two conserved PAPS binding motifs: 5’-PSB and 3’-PB (Supp_FigS2c). mWscd1 and mWscd2 belong to a unique clade different from other known glycan-specific sulfotransferases (Supp_FigS3a, Supp_DataS1). Orthologous genes are ubiquitously distributed in the deuterostomes from echinoderms to vertebrates (Supp_FigS3b, Supp_DataS2), which is consistent with the reported occurrence of SiaS in sea urchin^31–39^ and mammals^40–44^.

### Identification of Wscd1 and Wscd2 as SulT-Sias

To determine whether Wscd1 and Wscd2 were actually the SulT-Sias, the open reading frames of *mWscd1* and *mWscd2* were cloned into the pcDNA3.1-V5/His plasmid and used to transfect CHO cells. Forty-eight hours following transfection, both enzymes were detected at 71 kDa by western blotting with anti-V5 antibody (Fig. 3b,e), which coincided with their predicted molecular masses. The cell surface expression of SiaS was analyzed using flow cytometric analysis (FCA) with 3G9 (Fig. 3c,f). An apparent increase in 3G9-positive cells, as well as the % proportion of 3G9-positive cell population of the histograms, were observed for both *mWscd1*- and *mWscd2*-transfected cells compared to the mock cells (Fig. 3c,d,f,g). These results suggest that mWscd1 and mWscd2 have SulT-Sia activity. A previous study demonstrated that site-directed mutagenesis of the conserved 5’-PSB motifs (Fig. 3a) abolished sulfotransferase activity^47^. To confirm that mWscd1 and mWscd2 were SulT-Sias, alanine mutants within the 5′-PSB region were constructed (mutWscd1 and mutWscd2, respectively; Supp_FigS4) and expressed in CHO cells to determine the surface expression of SiaS (Fig. 3c,e). FCA revealed a significant decrease in the levels of 3G9 epitope for mutWscd1 and mutWscd2 (Fig. 3d,g). Knockdown experiments of *Wscd1* and *Wscd2* expression by RNA interference were also performed. Human embryonic kidney (HEK) cells expressing only *Wscd1* (Supp_FigS5a) and SK-N-SH cells expressing *Wscd2* (Supp_FigS5d) were transfected with the short hairpin (sh)Wscd1 and shWscd2 plasmids. HEK cells transfected with shWscd1, but not shMock, displayed decreased levels of *Wscd1* mRNA (Supp_FigS5b) and surface 3G9 epitope (Supp_FigS5c). Similarly, SK-N-SH cells transfected with shWscd2, but not shMock, displayed decreased levels of *Wscd2* mRNA (Supp_FigS5e) and surface 3G9 epitope (Supp_FigS5f). The findings indicate that Wscd1 and Wscd2 are SulT-Sia enzymes that are active in vivo.

### Intracellular localization of mWscd1 and mWscd2

**Figure 4:**
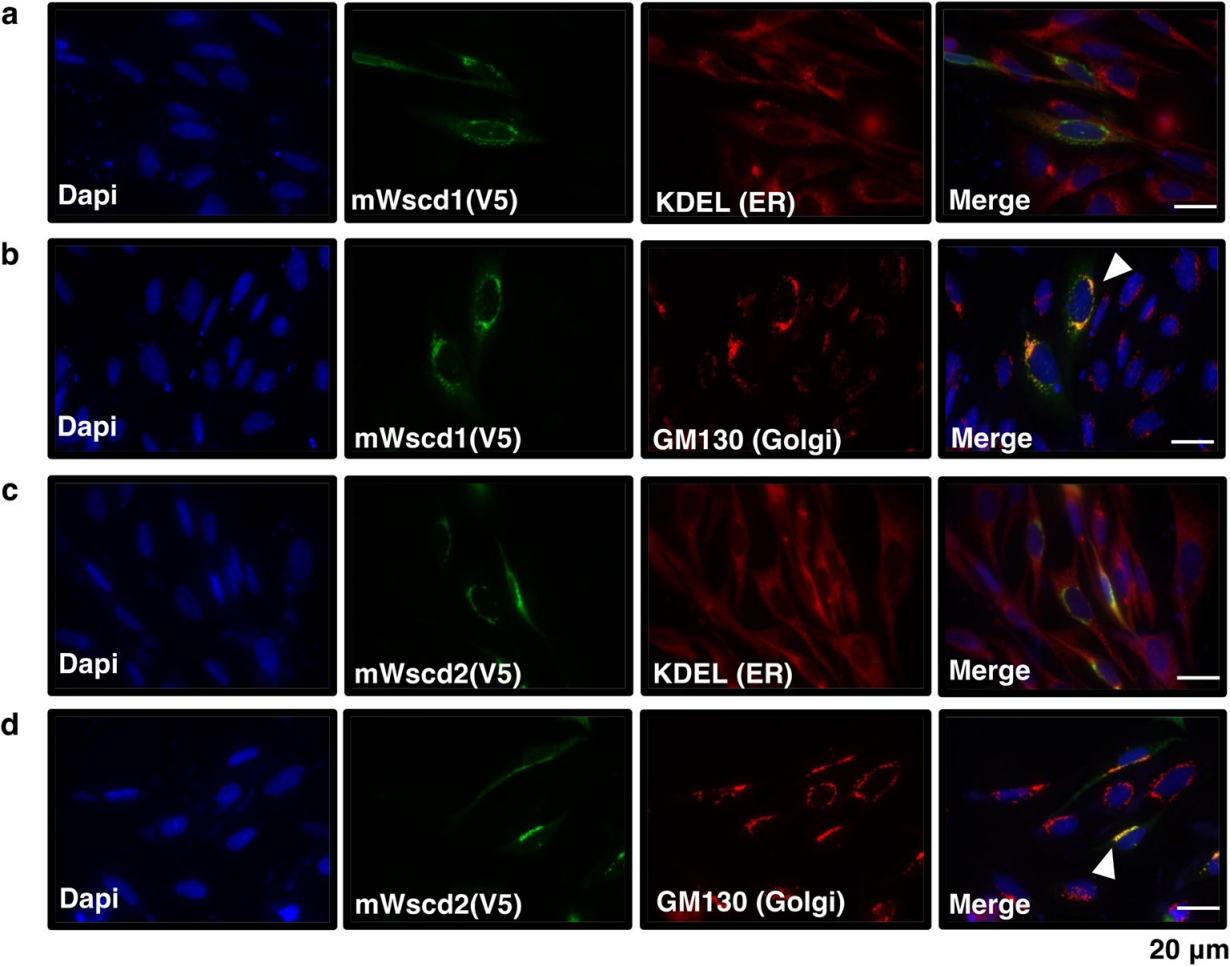
Intracellular localization of Wscd1 and Wscd2 in CHO cells. The V5-tagged Wscd1 and Wscd2 were immunostained with anti-V5 and Alexa-488-conjugated anti-chicken IgG (green) as the primary and secondary antibodies, respectively. Golgi apparatus is visualized with anti-GM130, followed by Alexa 555-conjugated anti-rabbit IgG (red). ER is visualized with anti-KDEL, followed by Alexa-594-conjugated anti-rabbit IgG (red). Nuclei are counter-stained with DAPI (blue). Co-immunostaining with anti-V5 and anti-GM130 (**a**, **c**), and anti-V5 and anti-KDEL (**b**, **d**) were performed.

Based on their predicted amino acid sequences (Fig. 3a), mWscd1 and mWscd2 were identified as type II transmembrane proteins with a short N-terminal cytosolic tail and a C-terminal catalytic domain. V5-tagged mWscd1 and mWscd2 were expressed in CHO cells. They were co-immunostained with GM130 but not KDEL (Fig. 4). Thus, they were Golgi-localized, as predicted.

### In vitro activity of mWscd1 and mWscd2

To test the in vitro sulfotransferase activity of mWscd1 and mWscd2, the recombinant enzyme fractions prepared from *mWscd1*-, *mWscd2*- and Mock-transfected CHO cells were incubated in 50 mM Tris–HCl, pH 7.2, at 20 °C for 18 h with 2 mM PAPS and the Sia-containing acceptor substrates Neu5Ac, cytidine-5′-monophosphate (CMP)-Neu5Ac, ganglioside GM1, and transferrin (TF). For Neu5Ac and CMP-Neu5Ac, no SiaS derivative was detected by the fluorometric HPLC analysis of the reaction mixture with Wscd1 or Wscd2 (data not shown). The findings indicated that sulfation of free Sia or donor substrate of sialyltransferases did not occur.

**Figure 5:**
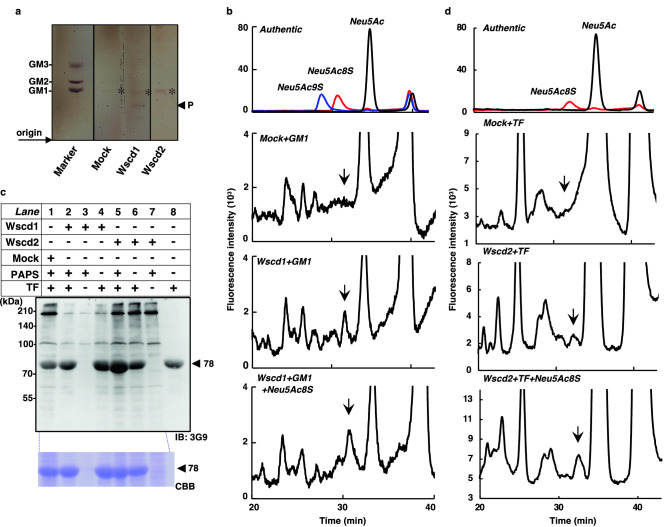
In vitro SulT-Sia activity of Wscd1 and Wscd2. (**a**) TLC of the products from the GM1 substrate incubated with the Wscd1, Wscd2, and Mock enzyme fractions, visualized by the orcinol reagent. The product P detected in the Wscd1 lane is marked. The bands denoted by asterisks are GM1. (**b**) Fluorometric HPLC of the product P in the Wscd1 lane in **a** (*Wscd1* + *GM1*) and the same area in the Mock lane in **a** (*Mock* + *GM1*). *Authentic*: Neu5Ac, Neu5Ac8S, and Neu5Ac9S; *Wscd1* + *GM1* + *Neu5Ac8S*: Co-injection of the DMB-derivatives in “*Wscd1* + *GM1*” with DMB-Neu5Ac8S. (**c**) SDS-PAGE/western blotting of the reaction mixtures containing the transferrin (TF) substrate and enzyme fractions using 3G9 (*upper panel*). Lane 1: TF with Mock + PAPS; lane 2: TF with Wscd1 + PAPS; lane 3: Wscd1 + PAPS; lane 4: TF with Wscd1; lane 5: TF with Wscd2 + PAPS; lane 6: TF with Wscd2; lane 7: Wscd1 + PAPS; lane 8: TF. *Lower panel*, CBB staining for TF in lanes 1–7 in the upper panel. The arrowhead at 78 kDa indicates TF; (**d**) Fluorometric HPLC profiles of DMB-derivatives for the TF-derived product by Wscd2 (*Wscd2* + *TF*). *Authentic*: Neu5Ac, and Neu5Ac8S; *Mock* + *TF*: TF incubated with Mock; *Wscd2* + *TF* + *Neu5Ac8S*: Co-injection of the DMB-derivatives in “*WSCD1* + *TF*” with DMB-Neu5Ac8S. The arrows in (**b**) and (**d**) denote the elution position of DMB-Neu5Ac8S.

For the glycolipid substrate, GM1 was incubated with the recombinant mWscd1, mWscd2, and Mock-derived enzyme fraction (Mock), and the reaction products were analyzed by thin-layer chromatography (TLC) (Fig. 5a). The bands denoted by the asterisk were detected for all reaction mixtures where the GM1 substrate was used, consistent with the migration rate of authentic GM1 control. On the other hand, the band denoted by P only appeared in the Wscd1 lane (Fig. 5a), suggesting that the activity of mWscd1 was specific for GM1. No band other than GM1 was detected in the Wscd2 lane (Fig. 5a), suggesting that GM1 was not the substrate of mWscd2. The band P in the Wscd1 lane and the same area in the Mock lane were then extracted for fluorometric HPLC analysis (Fig. 5b). The peak corresponding to the authentic Neu5Ac8S (Fig. 5b *authentic*) was detected in Wscd1 (Fig. 5b *Wscd1* + *GM1*), but not in the Mock fractions (Fig. 5b *Mock* + *GM1*). The co-injection experiment with authentic Neu5Ac8S also confirmed that the peak was Neu5Ac8S (Fig. 5b *Wscd1* + *GM1* + *Neu5Ac8S*). These results indicate that mWscd1 shows SulT-Sia activity on Neu5Ac residue on GM1, while mWscd2 has no activity against GM1.

The TF glycoprotein substrate, containing two *N*-glycan chains terminated with α2,6-Neu5Ac residues, was incubated with mWscd1, mWscd2, and Mock, and subjected to western blotting with 3G9 (Fig. 5c). TF was shown to contain the pre-existing 3G9 epitope (Fig. 5c *upper lane 8*); however, only when incubated with mWscd2 (Fig. 5c *upper lane 5*), but not mWscd1 (Fig. 5c *upper lane 2*) or Mock (Fig. 5c *upper lane 1*), the 3G9 epitope intensity was greatly increased in the TF band at 78 kDa. The amount of TF analyzed in each lane was the same based on the Coomassie Brilliant Blue (CBB) staining intensity of the same gel (Fig. 5c *lower*). In addition, this intensity increase did not occur in the absence of PAPS (Fig. 5c *upper lanes 4, 6*) or TF (Fig. 5c *upper lanes 3, 7*). These results indicate that Wscd2 shows SulT-Sia activity on TF, whereas Wscd1 has no effect on TF. The reaction product of Wscd2 was also analyzed to determine the increase in SiaS by fluorometric HPLC (Fig. 5d). The Neu5Ac8S peak was detected in the reaction product of Wscd2 (Fig. 5d *Wscd2* + *TF*) but not in Mock (Fig. 5d *Mock* + *TF*). The co-injection experiment also confirmed that the peak was Neu5Ac8S (Fig. 5d *Wscd2* + *TF* + *Neu5Ac8S*). Thus, Wscd2 shows SulT-Sia activity on the Neu5Ac residue of the TF *N*-glycan.

### Characterization of medaka Wscd1 and Wscd2

To gain insight into the significance of Wscd1 and Wscd2 in vertebrates at the organism level, we chose the medaka fish, *Oryzias latipes*, as a vertebrate model. Medaka has a single copy of each *Wscd1* and *Wscd2* orthologs genes. The *Wscd1* and *Wscd2* cDNAs were cloned from the fry at 6 days post-fertilization (dpf) and 7 dpf, respectively. Their deduced amino acid sequences showed 72% and 75% identity with those from mice, respectively (Supp_FigS6). Based on the real-time quantitative PCR (qRT-PCR) results, these genes were expressed in developing fry at least before hatching (9 dpf), especially after 2 dpf (Supp_FigS7a). They were also ubiquitously expressed in various organs of 3-month-old adult fish (Supp_FigS7b). Both genes were expressed at comparable levels in the kidney, eye, spleen, heart, intestine, and testis tissues, while the expression of *Wscd1* was dominant in the brain, liver, muscle, and ovary tissue, the latter being most prominent (Supp_FigS7b). These cDNAs were transfected and expressed in the CHO cells to investigate the SulT-Sia activity. Both enzymes exhibited SulT-Sia activity, based on the results from the FCA with 3G9 (Supp_FigS8). An apparent increase in the % proportion of 3G9-positive cell population of the histograms was observed for both medaka *Wscd1*- and *Wscd2*-transfected cells compared to the mock cells.

### Phenotypes of Wscd1- and Wscd2-deficient medaka

**Figure 6:**
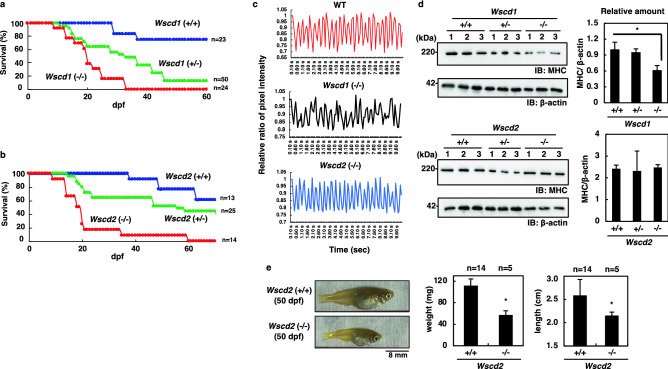
Phenotypes of the *Wscd1*- or *Wscd2*-deficient medaka. (**a**, **b**) Survival curves for (−/−), (+/−), and (+/+) medaka in *Wscd1* (**a**) or *Wscd2* (**b**) gene, respectively. In-cross offspring of the heterozygous genotype medaka were used. (**c**) Impaired heartbeat of *Wscd1*(−/−) fry at 8 dpf. The circulating blood cells amount in the ventricular chamber was monitored in wild-type (WT), *Wscd1*(−/−), and *Wscd2*(−/−) fry for 10 s, analyzed by the Image-J. (**d**) Reduction in the amount of myosin heavy chain (MHC) in *Wscd1*(−/−) fry at 8 dpf. (*Left panels*) Western blotting of *Wscd1*(+/−) (*upper panels*) or *Wscd2*(+/−) (*lower panels*) using antibodies against MHC and β-actin (loading control). (*Right panels*) Values for the MHC/β-actin ratio. The error bars indicate the standard deviations from three independent data. *p < 0.05. (**e**) Growth retardation of *Wscd2*(−/−) fry at 50 dpf. *Wscd2*(+/+) and surviving *Wscd2*(−/−) fry (*left*) were analyzed for the weight **(middle)**, and length **(right)**. The number of fish are indicated by n. The error bars indicate the standard deviations. *p < 0.05, (Student t-test, n = 3).

We generated Wscd1- and Wscd2-deficient medaka using the CRISPR-Cas9 system. The *Wscd1*-knockout medaka, *Wscd1*(−/−), had a 19-bp deletion in exon 2 (Supp_FigS9a) and the *Wscd2*-knockout medaka, *Wscd2*(−/−), had a 1-bp insertion/32-bp deletion in exon 1 (Supp_FigS9b). Since both knockout medaka strains could not grow into adult fish, the medaka strains with the heterozygous genotype [*Wscd1*(+/−) and *Wscd2*(+/−)] were established, and subcultured. The survival rate in the crossed offspring was investigated. *Wscd1*(−/−) fry showed lethality at 10 ~ 33 dpf with a dpf value of 50% death (D50) of 19.5 dpf (Fig. 6a). *Wscd2*(−/−) fry showed lethality at 9 ~ 60 dpf with a D50 of 18 dpf (Fig. 6b). Therefore, the *Wscd2*(−/−) fry lived a little longer than the *Wscd1*(−/−) fry, and 10% of *Wscd2*(−/−) fry could live until 60 dpf.

Notably, cardiac arrhythmia^48^ was observed in *Wscd1*(−/−) fry at 8 dpf and beyond (Fig. 6c). In these fries, circulating blood cells remained in the ventricular chamber for a longer time than in the wild-type (WT) fry, suggesting that the ventricular contractile force was weaker in *Wscd1*(−/−). Consistent with this result, the western blotting with an anti-myosin heavy chain (MHC) antibody (MF20)^49^ showed that the amount of cardiac MHC was significantly reduced in *Wscd1*(−/−) fry compared to the WT and *Wscd1*(+/−) fry (Fig. 6d *upper*). In contrast, *Wscd2*(−/−) fry did not develop cardiac arrhythmia (Fig. 6c), or any changes in MHC levels compared to the WT and *Wscd2*(+/−) fry (Fig. 6d lower). Taken together, these results indicate that *Wscd1*, but not *Wscd2*, is essential for heart development.

Although 50% of the *Wscd2*(−/−) fry died by 18 dpf, 8% lived as long as 60 dpf. Notably, *Wscd2*(−/−) fry at 50 dpf were smaller than the WT by 49% and 17% in weight and length, respectively (Fig. 6e). Even at 8 dpf, Wscd2(−/−) fry showed smaller eyes and brains than the WT, while *Wscd1*(−/−) fry did not (Supp_FigS10). These observations suggest that growth retardation might occur in *Wscd2*(−/−) fry, partly due to impaired muscle, eyes and brain development.

Interestingly, even heterozygous fry of *Wscd1*(+/−) and *Wscd2*(+/−) showed high lethality rates (84% and 43% compared to the WT, respectively; Fig. 6a,b). The remaining fry survived to maturity (approximately 90 dpf), and were fertile to provide the next generation. Considering that the heterozygous fry may express half the amounts of enzymes compared to the WT, the expression levels of Wscd1 and Wscd2 might critically affect the fry survival during the growth stages between 46 and 60 dpf. A notable feature of this period is that the survival curves showed a gradual, but not an acute, reduction (Fig. 6a,b). This result might be related to the severity of the inflammation states in these heterozygous fries. Since medaka fry were reared in non-sterile laboratory conditions, opportunistic infections were possible. Therefore, the inflammation states of fry at 15 dpf were investigated by monitoring the C-reactive protein (CRP) expression level, a marker of inflammation^50^, by qRT-PCR (Supp_FigS11). The *CRP* expression level was increased in *Wscd1*(+/−) fry compared to the WT or *Wscd1*(+/+) fry and was even higher in *Wscd1*(−/−) fry (Supp_FigS11*left panel*). The same or even more prominent results were obtained for *Wscd2*(+/−) and *Wscd2*(−/−) fry (Supp_FigS11*right panel*). Since CRP increases in response to inflammation^51^, the homozygous and heterozygous fry of both types are suggested to be in an inflammatory state, which is more severe in the homozygous than heterozygous fry. The findings further suggest that the loss of Wscd1 or Wscd2 increases the severity of inflammation in the fry, probably through the dysfunction of various tissues during the growth stages. Further studies are necessary to understand the linkage between the deficiency of these SulT-Sias and the inflamed state. The collective findings indicate the critical roles of Wscd1 and Wscd2 in the survival of medaka.

## Discussion

In conclusion, SiaS occurs in various cells and tissues in vertebrates including fish and mammals, and the sialate: *O*-sulfotransferases, Wscd1 and Wscd2, responsible for the sulfation of Sia residues on glycoproteins and glycolipids are constitutively expressed during embryogenesis and in various adult organs. Wscd1 and Wscd2 are structurally and phylogenetically close to each other, and this gene pair widely distributes in the deuterostomes from echinoderms to vertebrates. Interestingly, mWscd1 and mWscd2 may have complementary substrate preferences to each other, because mWscd1, but not Wscd2, is active for glycolipid GM1, while mWscd2, but not mWscd1, for glycoprotein transferrin. This feature might explain constitutive co-expression profiles of these two genes in embryos and adult organs of medaka. Both enzymes are Golgi-localized, type II transmembrane proteins with a short N-terminal cytosolic tail and a C-terminal luminal catalytic domain, and share the common structural and topological features with sialyltransferases^52,53^. Thus, sequential reactions of sialylation and sulfation may effectively proceed in the Golgi compartment.

**Figure 7:**
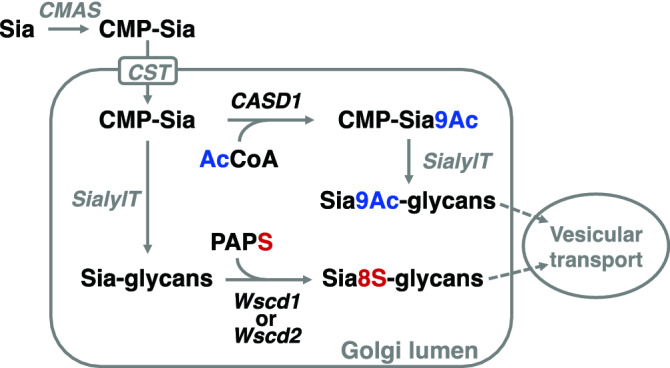
*O*-Sulfation and *O*-acetylation of Sia occur at different metabolite levels in the Golgi. For sialoglycans (Sia-glycans) biosynthesis, sialic acid (Sia) is first activated to CMP-sialic acid (CMP-Sia) by CMP-Sia synthetase (*CMAS*) in the cytosol and/or nucleus. CMP-Sia is then transported into the Golgi lumen by CMP-Sia transporter (*CST*), and used as a donor substrate of sialyltransferases (*SialylT*) to synthesize Sia-glycans on proteins and lipids. 9-*O*-Acetylation of Sia occurs at the CMP-Sia level using acetyl-coenzyme A (AcCoA) as a donor and 9-*O*-acetylated Sia (Sia9Ac) residue is synthesized by *SialylT* using CMP-Sia9Ac as a substrate. In contrast, 8-*O*-sulfation occurs at the Sia-glycan level using 3′-phosphoadenosine 5′-phosphosulfate (PAPS) as a donor, forming 8-O-sulfated Sia (Sia8S)-glycans. *CASD1* Cas domain containing 1, *Wscd1* Wsc domain containing 1, *Wscd2* Wsc domain containing 2.

Wscd1 and Wscd2 are the second examples of Sia modification enzymes, following the discovery of sialate: 9-*O*-acetyltransferase CASD1^29,30^. CASD1 converts CMP-Sia into CMP-Sia9Ac using acetyl-coenzyme A as a donor substrate^30^. Although both Sia modification enzymes are localized in the Golgi, Wscd1 and Wscd2 transfer sulfate group on sialoglycans on proteins and lipids, but not CMP-Sia, using PAPS as a donor substrate (Fig. 7). It may be concluded that Sia modifications occur in the Golgi compartment, although *O*-sulfation and *O*-acetylation of Sia residues occur at different metabolite levels before and after sialylation, respectively.

Examination of gene knockout medaka revealed that SiaS is essential at the organism level. *Wscd1*(−/−), but not *Wscd2*(−/−), fry at 8 dpf suffer from cardiac arrhythmia^48^, suggesting that *Wscd1* is essential for heart development. *Wscd2*(−/−), but not *Wscd1*(−/−), fry show growth retardation, accompanying impaired muscle, eyes, and brain development. Wscd2 might be involved in survival of cells in muscle, eye, and brain. Furthermore, the loss of Wscd1 or Wscd2 increases the severity of inflammation in the fry, which may be related to the lethality of fry. Although further studies are necessary to gain in-depth insight into pathophysiology of the lethality, the Sia: *O*-sulfation must have multiple functions such as embryonic development, organogenesis, and immune recognition. Finally, the present data emphasize the importance of modified Sias that have been uncovered for a long time.

Now that two different genes for SulT-Sia are evident, many questions would immediately emerge in various aspects of biochemistry and biology of SiaS. We can ask if there are still other genes for SulT-Sia. Recently, we reported that the surface expression of SiaS reversibly induced by treatment of CHO cells with the antibiotic G418^54^. Since CHO cells did not express Wscd1 or Wscd2 before and after the G418 treatment (unpublished results), the presence of other SulT-Sia than Wscd1 and Wscd2 is suggested in CHO cells. Thus, more sulfotransferases with the SulT-Sia activity might occur in mammalian cells. We can also seek for substrate specificity of the SulT-Sias in detail. This report shows that Wscd1 and Wscd2 can synthesize Neu5Ac8S-residues from Neu5Ac-residues; however, Sia species-specificity of the enzymes to Neu5Ac, Neu5Gc, and Kdn, which must be dependent on organism-species, and the linkage-specificity of sulfation not only to 8-*O*-position, but also to 4-*O*-, 7-*O*-, and 9-*O*-positions of Sia remains to be investigated. Last but not least, we should pursue a regulatory feature of SiaS expression through *O*-sulfation and de-*O*-sulfation like that of SiaAc expression through *O*-acetylation and de-*O*-acetylation^11,15,23^. To the best of our knowledge, no de-*O*-sulfating activity of SiaS has been identified so far, and this important aspect still remain an open question.

## Methods

### Materials

N-acetylneuraminic acid (Neu5Ac), cytidine-5′-monophosphateNeu5Ac (CMP-Neu5Ac), bovine serum albumin (BSA), and trifluoroacetic acid (TFA) were purchased from Nacalai Tesque (Kyoto, Japan). Ganglioside GM1 (Galβ1 → 3GalNAcβ1 → 4(Neu5Acα2 → 3) Galβ1 → 4Glcβ1-Cer) was purchased from AdipoGen (San Diego, CA, USA). 1,2-Diamino-4,5-methylenedioxy-benzene (DMB) was purchased from Dojindo Molecular Technologies, Inc. (Kumamoto, Japan). 3′-Phosphoadenosine 5’-phosphosulfate (PAPS) and 4′,6-diamidino-2-phenylindole (DAPI) were purchased from Sigma-Aldrich (Tokyo, Japan). Transferrin (TF) from humans was purchased from Wako (Osaka, Japan). TRI REAGENT LS was a product of Molecular Research Center, Inc. (Cincinnati, USA). pGEM-T Easy plasmid was purchased from Promega (Madison, WI, USA). The pcDNA3.1(neo) plasmid, pcDNA4-V5/His, and the mMessage mMachine SP6 transcription kits were purchased from Thermo Fisher Scientific KK (Tokyo, Japan). pSUPER.neo vector was a product of Oligoengine (Seattle, WA, USA). AmpliScribe T7-Flash Transcription kit was a product of Lucigen (WI, USA). ProtoScript II and BsaI were products of New England Biolabs (Ipswich, USA). The Cas9 expression vector with SP6 promoter, pCS2 + hSpCas9, and the sgRNA expression vector with a T7 promoter, pDR274, were gifts from Masato Kinoshita (Addgene 51815)^55^, and Keith Joung (Addgene 42250)^56^, respectively. Restriction enzymes, Ex Taq polymerase, and an In-Fusion HD Cloning Kit were purchased from TaKaRa Bio Inc. (Shiga, Japan). KOD SYBR qPCR mix (QKD-201), KOD-Plus-Neo polymerase and Bgl II were purchased from TOYOBO (Osaka, Japan). Polyvinylidene difluoride (PVDF) membranes and enhanced chemiluminescence (ECL) reagents were purchased from (GE Healthcare, Tokyo, Japan). Molecular weight markers and bicinchoninic acid (BCA) assay kits were purchased from Bio-Rad (Hercules, CA, USA). PEI-Max Transfection Reagent was a product of Polysciences Inc. (PA, USA). Anti-β-actin antibody was purchased from Santa Cruz Biotechnology (Dallas, TX, USA). Peroxidase-conjugated goat anti-mouse (IgG + IgM) was purchased from American Qualex (San Clemente, CA, USA). The monoclonal anti-myosin 4 antibody (MF20), Alexa Fluor 488-labeled goat anti-mouse IgM, and Alexa Fluor 488-labeled goat anti-mouse (IgG + IgM) antibodies were obtained from Thermo Fisher Scientific KK. Alexa Fluor 488-conjugated anti-chicken IgY, anti-GM130, and anti-KDEL antibodies were products of Abcam (Cambridge, UK). Alexa Fluor 555-conjugated anti-rabbit IgG was purchased from Invitrogen (Carlsbad, CA, USA). Chicken anti-V5 antibody was purchased from Bethyl laboratories (Montgomery, TX, USA). 4-Methylumbelliferyl ⍺-glycoside of 8-*O*-sulfated Neu5Ac (4MU-Neu5Ac8S) was prepared as previously described^45^. The monoclonal IgM antibody 3G9 (3G9), which specifically recognizes the α-glycosides of Neu5Ac8S, was previously prepared using sea urchin sperm as an immunogen^45,46^. mAb.2G9 (10 μg/mL) was obtained as an IgM clone, which did not react with Neu5Ac8S-containing gangliosides, during the 3G9 preparation and was used as the isotype control. Unless otherwise stated, primers for PCR amplification were purchased from RIKAKEN (Nagoya, Japan).

### Ethics statement and the ARRIVE guidelines

All procedures for the use of animals were approved by the Animal Care and Use Committee of Nagoya University (Permit Number: BBC2019001 for medaka; BBC2019002 for mouse), and performed under the relevant Animal Research: Reporting of In Vivo Experiments (ARRIVE) guidelines and regulations, which are set by the same committee. All methods were performed in accordance with the relevant guidelines and regulations. Every effort was made to minimize the number of animals used and their suffering.

### Animals

Mice (C57/BL6J) were obtained from Japan SLC (Hamamatsu, Japan) and brain samples were prepared at different stages: embryo 14.5 days (E14.5), postnatal day 1 (P1), P15, P30, and P45. The Nagoya strain of medaka fish, *Oryzias latipes*, was used as the wild type (WT). Wild type medaka strain was supplied by the National Bioresource Project (NBRP) Medaka. Fish stocks were maintained in 16-L tanks with a circulating water system maintained at 26 °C and a 14 h light/10 h dark cycle. The development and phenotype of medaka fish were observed using an SZX12 DP80 microscope (Olympus, Tokyo, Japan).

### Immunohistochemistry of tissue sections

Mouse tissue section slides (Normal organs of adult mice ZE1, 8-week-old, ICR strain) were purchased from Super Bio Chips (Seoul, Korea) and human tissue section slides (BA4 evaluation slide) were obtained from Gentaur Molecular Products (Kampenhout, Belgium). According to the manufacturer’s instruction, these sections were 4% formaldehyde-fixed paraffin-embedded, 4 mm-thick section on the glass slide. The tissue sections on the slide were rehydrated, proteinase K-digested (5 mg/mL, 50 mM Tris–HCl, pH 7.5, 5 mM EDTA) at room temperature (rt) for 10 min, followed by treatment with 3% H_2_O_2_ for 6 min. After washed three times, they were blocked with 2% BSA in 10 mM sodium phosphate buffer, pH 7.2, 0.15 M NaCl (PBS) for 30 min, and incubated with 1 µg/mL 3G9 at 4 °C for overnight. After washed, they were incubated with Alexa Fluor 488-labeled goat anti-mouse IgM (1:500 dilution) for 30 min. Nucleus was stained with 1 µg/mL DAPI for 15 min. The immunofluorescence was observed under the fluorescent microscopy BX51 (Olympus, Tokyo, Japan)^3,45^.

### SDS-PAGE and western blotting

Cells and tissues were homogenized with the lysis buffer containing 1% Triton X-100, 1 mM phenylmethylsulfonyl fluoride (PMSF), protease inhibitor cocktail (1 μg/mL each of aprotinin, leupeptin, and pepstatin, 2 μg/mL of antipain, and 5 mM EDTA), phosphatase inhibitor cocktail (50 mM sodium fluoride (NAF), 10 mM β-glycerophosphate, and 1 mM sodium *o*-vanadate) in PBS and incubated on ice for 1 h. Homogenates were centrifuged at 15,000 × *g* at 4 °C for 15 min. The total protein concentration of the supernatants was quantified using the BCA assay. Each lysate was denatured with 5% (v/v) β-mercaptoethanol at 60 °C for 20 min to detect SiaS and then subjected to 6% SDS-PAGE, followed by blotting onto a PVDF membrane using a semi-dry blotting apparatus^39,45^. The PVDF membrane was blocked with 2.5% dry milk in PBS containing 0.05% Tween 20 (PBST) at 37 °C for 1 h. Incubation with the primary antibody (2 µg/mL 3G9) was performed in PBST containing 2.5% dry milk at the 4 °C overnight. After washing three times with PBST, the membrane was incubated with peroxidase-conjugated anti-mouse IgG + M as a secondary antibody (1: 5000 dilution) at 37 °C for 1 h. After washing three times with PBST, the ECL reagents were used to visualize the blotted components.

### Fluorometric high performance liquid chromatography (HPLC) analysis

To assess the amount of SiaS components of the glycoproteins blotted onto PVDF membranes^57^, E14.5 mouse brain homogenates (100 µg) were applied to 10% SDS-PAGE followed by blotting on a PVDF membrane. The left edge of the membrane was removed and used for western blotting with 3G9. The rest of the membrane was equally cut into nine pieces according to the molecular size. Each slit was cut into small pieces and subjected to mild acid hydrolysis in 0.4 mL of 0.1 N TFA at 80 °C for 2 h. The hydrolysates were dried using SpeedVac vacuum concentrator (Savant, Thermo Fisher Scientific). Twenty microliters each of 0.01 M TFA and DMB solution^39,45^ were added and incubated at 50 °C for 2 h. The DMB-derivatized samples were directly applied to an octadecylsilyl (ODS) column (250 × 4.6 mm i.d., Capcellpak C18 type MG, Shiseido, Tokyo, Japan) and eluted with acetonitrile/methanol/0.05% TFA (4:6:90, v/v/v) at 1.0 mL/min for 120 min on a JASCO HPLC system (excitation, 373 nm; emission, 448 nm) as previously described^45^. 4MU-Neu5Ac8S was used as a positive control. For identification of DMB-derivatives of Neu5Ac and Neu5Ac8S, the identical retention times to the authentic sialic acid species were confirmed. In addition, co-injection experiments of the samples and CMP-Neu5Ac or CMP-Neu5Ac8S were also performed.

### Cloning of Wscd1 and Wscd2 cDNA from mice and medaka

The cDNAs for the mouse cell wall integrity and stress response component (WSC) domain containing 1 (*mWscd1*) and *mWscd2* genes (Gene ID: 216881 and 320916, respectively) were obtained by amplifying the coding regions by PCR using specific primers (Table S1) and Ex Taq DNA polymerase according to the manufacturer’s protocol. Total RNA was prepared from mouse embryonic brain (E14.5) using TRI REAGENT LS. First strand cDNA was synthesized using random hexamer primers from 1 μg of total RNA as the template using ProtoScript II reverse transcriptase. The PCR conditions were 30 cycles of a step program (94 °C for 1 min, 55 °C for 30 s, and 72 °C for 1 min). The product was cloned into the pGEM-T Easy plasmid. DNA sequences were analyzed using the deoxynucleotide chain termination method. The cDNAs for medaka *Wscd1* (*mdkWscd1*) and *mdkWscd2* genes (gene ID: 101157150 and 101164728, respectively) were obtained by amplifying the coding regions through PCR using the primary cDNA prepared from 6-dpf and 7-dpf fry, respectively, primers (Table S1), and Ex Taq DNA polymerase. The PCR products were also cloned into the pGEM-T easy plasmid.

### Molecular phylogenetic analysis

Nucleotide sequences of genes that are annotated as mouse sulfotransferases or Wscd1/Wscd2 of various animals were obtained from the National Center for Biotechnology Information (NCBI) gene database (https://www.ncbi.nlm.nih.gov/gene/). Multiple sequence alignment of all the sequences was performed by ClustalW 2.1 (DNA Matrix; IUB, Slow Pairwise Alignment, Tossgaps) and the phylogenic tree was obtained by the Neighbor-joining method (Kimura method) on GENETYX software Ver.14.

### Plasmid preparations

(a) Mammalian expression plasmids for Wscd1 and Wscd2. The cDNA fragments were amplified by a two-step cycle PCR (98 °C for 10 s, 68 °C for 1 min, 30 cycles) with KOD-Plus-Neo polymerase from the *mWscd1*- and *mWscd2*-encoded pGEM-T easy plasmids using the primers with an additional 15 bp at both 5′- and 3′-ends that matched the linearized pcDNA3.1-V5/His plasmid (Table S2), and subcloned into the pcDNA3.1-V5/His using an In-Fusion HD Cloning Kit. The *mdkWscd1*-, and *mdkWscd2*-encoded pcDNA plasmids were prepared by the same procedures, except that the corresponding pGEM-T easy plasmids and primers (Table S2), and pcDNA4-Myc/His plasmid were used. The obtained plasmids were denoted pcDNA-mWscd1, pcDNA-mWscd2, pcDNA-mdkWscd1, and pcDNA-mdkWscd2, respectively. The integrity of all plasmids was confirmed by DNA sequencing using the dideoxynucleotide chain termination method. (b) Plasmids for mutWscd1 and mutWscd2: To obtain an inactivated form of mWscd1 or mWscd2, and mutWscd1 or mutWscd2, the PAPS binding domain sequence (357–363 or 356–362 amino acids, respectively) was impaired (Supp_FigS5). Four critical amino acid residues (proline-357 or 356, glycine-360 or 359, threonine-362 or 361, and tryptophan-363 or 362 for mutWscd1 or mutWscd2, respectively) in the conserved PAPS binding domain were replaced with alanine residues. A sequential site-directed mutations of pcDNA-mWscd1 or pcDNA-mWscd2 (see above) were performed to obtain plasmids containing the four-amino acid-mutated *mWscd1* or *mWscd2* genes, respectively. The PCR conditions, templates, and primers used are described in Table S3. In each procedure, two-step cycle PCR (98 °C for 10 s, and 68 °C for 4 min; 30 cycles) was performed. The product was digested with DpnI to remove the remaining template plasmid. DH5⍺ cells were transformed with the DpnI-digested product (1 μL). The integrity of the plasmid was confirmed by DNA sequencing. (c) Short hairpin RNA (shRNA) plasmids: The shRNA plasmids were prepared using the pSUPER.neo vector according to the manufacturer’s instructions. Sense and antisense oligonucleotides for suppressing the expression of human *Wscd1* or *Wscd2* gene (ID: 23302 or ID: 9671, respectively) were designed using the siDirect tool (http://sidirect2.rnai.jp), and purchased from Eurofins Japan (Luxembourg, Netherlands) (Table S4). They were heated at 94 °C for 4 min in 100 mM NaCl, 10 mM Tris–HCl, pH 8.0, 1 mM EDTA, annealed by slowly cooling to 20 °C in steps of 2 °C every 4 min, and ligated into the pSUPER.neo vector by T4 DNA ligase to obtain the shWscd1 and shWscd2 shRNA plasmids. As a control, the pSUPER.neo plasmid was used as shMock instead of shWscd1 or shWscd2. DH5⍺ cells were transformed with the Bgl II-digested product (1 μL). The integrity of the plasmid was confirmed by DNA sequencing. (d) pDR274 plasmid with sgWscd1 or sgWscd2 sequence: Construction of the pDR274 plasmids encoding the CRISPR-Cas9 targets was previously described^58^. Briefly, target sequences in sgRNAs for medaka *Wscd1* or *Wscd2* gene were designed using their sequences, ENSORLG00000000526 and ENSORLG00000006858, respectively, at the Ensembl Genome Database Project. Oligonucleotide pairs containing the target sequences (Table S5) were synthesized by Hokkaido System Science Co., Ltd. (Hokkaido Japan). They were heated in 40 mM Tris–HCl, pH 8.0, 20 mM MgCl_2_, and 50 mM NaCl at 95 °C for 2 min and annealed by cooling slowly to 25 °C in 1 h (0.1 °C /s). The annealed oligonucleotides were then ligated into the BsaI-digested pDR274 vector. The obtained plasmids were denoted pDR274-sgWscd1 and pDR274-sgWscd, respectively.

### Cell culture

Chinese hamster ovary (CHO) and human embryonic kidney (HEK) cells were purchased from Riken Cell Bank (Tsukuba, Japan). The human neuroblastoma (SK-N-SH) cell line was purchased from the Japanese Collection of Research Bioresources Cell Bank (Kobe, Japan). CHO and SK-N-SH cells were cultured in MEM-α (Wako, Japan) supplemented with 100 U/mL penicillin G and 100 μg/mL streptomycin sulfate, and 10% fetal bovine serum in a 5% CO_2_ and 95% air-humidified atmosphere at 37 °C. HEK cells were cultured under the same conditions except that Dulbecco's modified Eagle's medium was used instead of MEM-α.

### Cell transfection

CHO cells (0.5 × 10^6^ cells) were cultured in a 6-well plate overnight at 37 °C, and transiently transfected with 3 μg of each pcDNA3.1 plasmid for mWscd1 and mWscd2, and pcDNA4 plasmids for mdkWscd1 and mdkWscd2 using the PEI-Max Transfection Reagent. At 48 h post-transfection, the cells were collected and subjected to flow cytometry and fluorometric HPLC analyses to evaluate the SiaS epitope. Transfection efficiency was evaluated by observing the transfected cells with a plasmid encoding the green fluorescent protein (GFP) gene by fluorescent microscopy. For RNA interference with shRNA plasmids, transfection of HEK and SK-N-SH cells was performed with the shWscd1 and shWscd2 plasmids, respectively, according to the above-mentioned method.

### Flow cytometry analysis (FCA)

Analysis of cell surface SiaS expression was performed by FCA. Cells were collected with a cell scraper, washed twice with PBS, and blocked with 5 mM EDTA and 0.5% BSA at 4 °C for 30 min. They were incubated with 3G9 (10 µg/mL) at 4 °C for 1 h, and washed twice with PBS. mAb.2G9 (10 μg/mL) was used as an isotype control. The cells were incubated with Alexa Fluor 488-labeled goat anti-mouse (IgG + IgM) (2 µg/mL) at 4 °C for 30 min. After washing twice with PBS, the cell surface fluorescence was analyzed using a Galios flow cytometer (Beckman Coulter, Brea, CA, USA). The collected data were analyzed using the Kaluza software (Beckman Coulter). The proportion of the 3G9 epitope-positive to negative cells was analyzed.

### Subcellular localization

CHO cells (0.5 × 10^6^) were cultured on glass coverslips in 6-well plates until 60% confluency. The cells were then transfected with 3 μg of pcDNA-mWscd1, pcDNA-mWscd2, and pcDNA3.1-Mock (empty) plasmids. All cells were washed once with PBS and fixed by incubation with 4% paraformaldehyde in PBS at room temperature for 8 min. The membrane was permeabilized with 0.1% Triton-X-100 in PBS at room temperature for 15 min. The cells were blocked with 2% BSA in PBS for 1 h. V5-tagged Wscd1 and Wscd2 were stained by incubation with anti-V5 chicken mAb (1:500 dilution) at 37 °C for 1 h, followed by incubation with a 1:1000 dilution of Alexa Fluor 488-conjugated anti-chicken IgY at room temperature for 30 min. Golgi apparatus and endoplasmic reticulum were stained with GM130 mAb and KDEL mAb at 37 °C for 1 h, followed by incubation with Alexa Fluor 555-conjugated anti-rabbit IgG (1:1000 dilution). DAPI was used for nuclear staining at 37 °C for 15 min. The stained slides were examined using a KEYENCE BZ-X810 florescent microscope.

### In vitro sulfotransferase activity assay

(a) In vitro enzyme reactions: CHO cells (1.5 × 10^6^ cells) were inoculated in a 10-cm dish overnight, transfected with 3 μg of pcDNA-mWscd1, -mWscd2, or -Mock and incubated for 2 days. The transfected CHO cell lysate was used as the crude enzyme fraction for the in vitro activity. The reaction mixtures (50 μL) containing 50 mM Tris–HCl, pH 7.2, 0.375 mM 1,4-dithiothreitol, 2 mM ATP, 5 mM NAF, 10 mM MnCl_2_, 2 mM PAPS, Sia-containing acceptor substrates (TF, 2.5 µg or GM1, 10 nmol), and 25 µg of the enzyme fraction were incubated at 20 °C for 18 h. (b) GM1 substrate: The reaction mixtures with GM1 were mixed with 1 mL of chloroform/methanol/water (30:60:8, v/v/v) (CMW), and applied to a DEAE-Sephadex A-25 column (2.6 × 15 cm; preequilibrated with CMW). The column was washed with 1 mL of CMW, and eluted with 12.5 mL of chloroform/methanol/1 M CH_3_COONa (30:60:8, v/v/v) to obtain the acidic glycolipid fraction. After desalting with the SepPak C18 cartridge (Waters Corp, Milford, MA, USA), the acidic lipid fraction was spotted on a thin-layer chromatography (TLC) plate (Silica Gel 60, GE Healthcare), and developed with chloroform/methanol/0.2% CaCl_2_ (60:25:4, v/v/v). The TLC sheet was cut into two parts. One part was visualized for detecting the acidic glycolipid products (P) by the orcinol/sulfuric acid method^33,59^. The other part was used to collect the product P by scratching the silica gels at the corresponding position of P on the visualized plate. The product P was extracted from the collected silica gels by CMW, and the supernatant was subjected to fluorometric HPLC after hydrolyzed in 0.02 mL of 0.1 N TFA at 80 °C for 2 h. (c) TF substrate: The reaction mixtures with and without TF were subjected to SDS-PAGE/western blotting with 3G9 as described above. CBB staining was performed to check for equal substrate loading in the western blotting. The reaction products were also analyzed by the fluorometric HPLC, after hydrolyzed in 0.1 N TFA.

### Quantitative RT-PCR (qPCR)

Total RNA was extracted from cells and tissues by using TRI REAGENT LS. The amount of extracted RNA was quantified using a spectrophotometer, and the purity of RNA was checked by the ratio of absorbance at 260 nm and 280 nm. Total RNA (5 μg) was subjected to reverse transcription using ProtoScript II with a random hexamer primer. qRT-PCR was performed using a pair of oligonucleotides (Table S6) and SYBR GreenER qPCR Supermix for iCycler premix. Amplification was performed using the iCycler IQ real-time PCR analysis system (Bio-Rad). Gene expression profile modulations were assessed by comparing the Ct values using the 2^−∆∆Ct^ method. The medaka actin gene was used to normalize the expression of the genes of interest. All experiments were conducted in triplicate.

### Generation of Wscd1- and Wscd2-deficient medaka

All the procedures followed the instruction protocol by the NIBB55 (https://shigen.nig.ac.jp/medaka/)^58^. After pCS2 + hSpCas9 was linearized by NotI digestion, the vector was used as a template for the synthesis of capped Cas9 mRNA with an mMessage mMachine SP6 kit and then purified using the RNeasy Mini Kit. For the synthesis of sgRNAs, the pDR274 vector containing sg*Wscd1* or sg*Wscd2* was first digested by DraI. This was use as the template for synthesizing sgRNA using the AmpliScribe T7-Flash Transcription kit. The sgRNAs were purified using the RNeasy Mini kit. Approximately 2–4 nL of a mixture containing 100 ng/µL of Cas9 mRNA and 25 ng/µL of sgRNA of *Wscd1* or *Wscd2* was injected into embryos at the one-cell stage.

### Genotyping

The fin clips of selected medaka fish or larvae were fixed in 40 μL of methanol and lysed in an appropriate amount of protease K solution (10 mM Tris–HCl, pH 7.5, 10 mM EDTA, and 2 mg/mL proteinase K). It was then incubated at 55 °C for 3 h, followed by denaturation at 95 °C for 15 min to inactivate the protease K. After centrifugation, the supernatant of each sample was used as genomic DNA. To detect CRISPR/Cas9-induced mutations, a heteroduplex mobility assay (HMA) was performed as described previously^60–62^. The mutations were then sequenced using an appropriate primer set (Table S7).

### Assessment of survival rate of medaka

The G0 medaka were out-crossed with the wild-type medaka to obtain the F1 hetero-mutant medaka. The F1 hetero-mutants were subjected to genotyping to find Wscd1(+/−) and Wscd2(+/−) medaka, which contained a knockout allele arisen from frameshifts. The Wscd1(+/−) or Wscd2(+/−) female and male medaka of the same genotype were in-crossed with each other to obtain Wscd1(−/−) or Wscd2(−/−) offspring at F2. The Wscd1(+/−) or Wscd2(+/−) female and male medaka were carefully maintained as the stable strain containing the knockout allele. Their offspring that should contain all the (−/−), (+/−) and (+/+) genotypes were daily collected and maintained in a separate plastic rearing tank under a 14 h-day/10 h-night cycle at 26 °C. For each group, at least 5 small tanks were established to assess the life span. To understand the life span, the medaka were observed every day for a certain period. When some of them died, the dead fish were immediately collected for genotyping.

### Digital video recording and analysis of the heart contraction

Young fry from 3 to 9 dpf (hatching day) was immobilized in a hole made by 1.5% agarose. The heart contractions were recorded using an SZX12 DP80 microscope (Olympus). Digital pictures were captured at maximum frame rate at a resolution of 1360–1024 pixels for up to 3 min and recorded in a PC using CellSens Standard software. Movies of heart movements were processed using ImageJ software. Contraction rhythms were measured based on alterations in the intensity of blood cell flow into and out of ventricle. Regions of interest (ROIs) in the ventricle were selected. The pixel intensities of the ROIs were digitized throughout the entire time series examined using ImageJ software.

### Growth assessment of WT and Wscd2(−/−)

Young fry of Wscd2(−/−) that were alive at 60 dpf and WT fry were measured for the body weight and body length as described previously^63,64^. Briefly, after anesthetized, the fish were weighed, and the body length was measured using the ruler. Five Wscd2(−/−) fish and 15 WT fish were used.

### Expression level of C-reactive protein (CRP) in 15 dpf young medaka

Expression level of *CRP* in WT, *Wscd1*(−/−), and *Wscd2*(−/−) fry at 15 dpf was determined by qRT-PCR using the primary cDNA as a template and the primers for *CRP* and *β-actin* (Table S6). *β-actin* expression was used as the housekeeping protein control. The *CRP*/*β-actin* ratios were calculated for each of the (+/+), (+/−), and (−/−) genotypes of *Wscd1* and *Wscd2*.

### Statistics

All values were expressed as the mean SE (n is three) and p-values were evaluated by the Student’s t-test.

## Supplementary Information

Supplementary Information.

## Data Availability

The nucleotide sequences reported in this paper will appear in the DNA Data Bank Japan (DDBJ) nucleotide sequence databases with LC669910 for mouse *Wscd2*; LC669911 for medaka *Wscd1*; and LC669912 for medaka *Wscd2*. Enter the ID at the DDBJ site: http://getentry.ddbj.nig.ac.jp/top-e.html.
