# Supplementary material for: Sulfation of sialic acid is ubiquitous and essential for vertebrate development
Source: Sci Rep. 2022 Jul 21;12:12496. doi: 10.1038/s41598-022-15143-4 (PMC9304399; doi:10.1038/s41598-022-15143-4)
Supplement: Supplementary file 1 — Supplementary Information. [file 41598_2022_15143_MOESM1_ESM.docx]

Supplementary Materials

**Sulfation of sialic acid is ubiquitous and essential for vertebrate development**

Nursah Ertunc^1,3^, Thanyaluck Phitak^1,4^, Di Wu^1,2^, Hiroshi Fujita^1^, Masaya Hane^1,2^,

Chihiro Sato^1,2^, and Ken Kitajima^1,2*^

^1^Bioscience and Biotechnology Center, and Graduate School of Bioagricultural Sciences, Nagoya University, Nagoya 464-8601, Japan.

^2^Institute for Glyco-core Research (iGCORE), Nagoya University, Nagoya 464-8601, Japan.

^3^Present address: Molecular Cell Biology, Faculty of Medical Technology, Graduate School of Health Sciences, Fujita Health University, 1‑98 Dengakugakubo, Kutsukake, Toyoake, Aichi  470‑1192, Japan.

^4^Present address: Biochemistry Department, Faculty of Medicine, Chiangmai University, Chiangmai 50200, Thailand.

*Correspondence to: Ken Kitajima, [kitajima@agr.nagoya-u.ac.jp](mailto:kitajima@agr.nagoya-u.ac.jp).

**-------------------------------------------------------------------------**

The supplementary information includes 11 figures, 8 figures for uncropped data, 7 tables, and 2 supplementary data.

Supp_ Figs. S1 to S11

Supp_Figs. S12 to S19 for uncropped data

Tables S1 to S7

Supplementary Data 1 to 2 (Separate Excel files)

**Legends for Supplementary Figures:**

Supp_FigS1. Immunohistochemistry of human tissue sections with 3G9. Sections of adult human tissues obtained from the kidney, liver, brain, breast, skin, and prostate were immunostained with 3G9 and 2G9 (isotype control). The SiaS epitopes were visualized by Alexa-488-conjugated anti-mouse IgM (Alexa, green). Nuclei were stained with DAPI (Dapi, blue).

Supp_FigS2. Amino acid sequences for mWscd1 and mWscd2. (a) RT-PCR of the full-length of *mWscd1* and *mWscd2* cDNAs. The first-strand DNA was prepared from the total RNA extracted from the E14.5 mouse embryonic brain. The *mWscd1* and *mWscd2* cDNAs were amplified from the first-strand DNA using the gene-specific primers (Supplementary Table 1); (b) Amino acid sequence alignments of mouse Wscd1 (mWscd1) and mWscd2. The sequences were deduced from the nucleotide sequences of cDNAs for mouse *Wscd1* and *Wscd2*. They contain two conserved PAPS binding motifs, 5’-PSB and 3’-PB in order (red underline). Accession numbers are Gene ID: 216881 for *Wscd1* and DDBJ LC669910 for *Wscd2*. The mWscd2 cDNA contained three silent mutations (57G>C, 954T>C, and 1125T>C) in Gene ID: 320916; (c) Putative PAPS binding sites in *Wscd1* and *Wscd2*. The *upper panels* display the consensus sequences of two conserved PAPS binding sites, 5’-PSB and 3’-PB, responsible for the binding to the 5’-phosphosulfate group and 3’-phosphate group pf PAPS, respectively, among sulfotransferases [65]. The *lower panel* displays the sequence alignment of mWscd1 and mWscd2 with 5’-PSB and 3’-PB of the mouse estrogen sulfotransferase (mES, marked by *). Red letters in the sequence represent identical amino acid residues of the three.

**Supp_FigS3. Molecular phylogenetic analysis of *Wscd1* and *Wscd2*.** (**a**) Phylogenetic tree of mouse sulfotransferase genes. The tree was generated based on the nucleotide sequences by the neighbor-joining method on Genetyx software. Wscd1 and Wscd2 are highlighted by squares. The Genebank accession numbers are listed in Supplementary Data 1. (**b**) Phylogenetic tree of Wscd1 and Wscd2 in deuterostome. The orthologous genes are ubiquitously distributed in deuterostome. *Homo sapiens*, human; *Pan troglodytes*, chimpanzee; *Gorilla gorilla gorilla*, gorilla; *Macaca mulatta*, rhesus monkey; *Mus musculus*, mouse; *Rattus norvegicus*, Norway rat; *Gallus gallus*, chicken; *Anolis carolinensis*, Green anole; *Xenopus laevis*, clawed frog; *Danio rerio*, zebrafish; *Oryzias latipes*, medaka fish; *Branchiostoma floridae*, lancelet; *Ciona intestinalis*, vase tunicate; *Saccoglossus kowalevskii*, acorn worm; *Strongylocentrotus purpuratus*, purple sea urchin. The phylogenetic tree was generated based on the amino acid sequences by the neighbor-joining method usiing Genetyx software. The Genebank accession numbers are listed in Supplementary Data 2.

Supp_FigS4. Structures of mutWscd1 and mutWscd2. The nucleotide and amino acid sequences of the 5’-PSB regions for Wscd1, Wscd2 and their Ala-mutants, mutWscd1 and mutWscd2, respectively.

Supp_FigS5. Effects of knockdown of the Wscd1 and Wscd2 genes. (a) RT-PCR of Wscd1, Wscd2, and β-actin genes in human HEK cells; (b) Expression level of Wscd1 mRNA of HEK cells transfected with shMock or shWscd1 plasmid, as measured by qRT-PCR; (c) FCA of the surface expression of SiaS with 3G9 on HEK cells transfected with shMock or shWscd1 plasmid. (*left* and *middle*) Mock-cells (shMock) and Wscd1-suppressing cells (shWscd1). The proportions of the 3G9-positive cell population (% area) are shown in each panel; (*right*) % Area obtained from the FCA histograms in *left* and *middle* panels. All the experiments were triplicated, and the error bars indicate the standard deviations. *p < 0.05 (Student t-test, n=3). (d) RT-PCR of Wscd1, Wscd2, and β-actin genes in human SK-N-SH cells; (e) Expression level of Wscd1 mRNA of SK-N-SH cells transfected with shMock or shWscd1 plasmid, as measured by qRT-PCR; (f) FCA of the surface expression of SiaS with 3G9 on SK-N-SH cells transfected with shMock or shWscd1 plasmid. See the legend for the *left,* *middle*, and *right* panels. All the experiments were triplicated, and the error bars indicate the standard deviations. *p < 0.05 (Student t-test, n=3).

Supp_FigS6. Deduced amino acid sequences for medaka Wscd1 and Wscd2. The cDNAs for mdk*Wscd1* and mdk*Wscd2* are cloned from the medaka fry at 6 dpf and 7 dpf, respectively. The cDNA sequence for mdkWscd1 (LC669911) contained four silent mutations (701A>G, 1452A>G, 1497T>C, and 1652G>A) and two missense mutations (59T>C, and 311A>G) in Gene ID:101157150, and that for mdkWscd2 (LC669912) contained four silent mutations (564T>C, 1029C>T, 1080T>C, and 1128T>C) and two missense mutations (928T>G, and 1090G>T) in Gene ID:101164728. mdkWscd1 and mdkWscd2 consist of 1695 and 1686 bp encoding 565 and 562 amino acid residues, respectively. These two amino acid sequences show 45 % identities with each other and contain two conserved PAPS binding motifs, 5’-PSB and 3’-PB (red underline).

Supp_FigS7. Expression profiles of *mdkWscd1* and *mdkWscd2* genes. (a) Developmental expression in pre-hatching stage fry. (b) Organ-dependent expression in 3-month-old-adult. The expression levels are quantified by qRT-PCR, and normalized to that of the housekeeping β-actin in the same sample. The error bars represent standard deviations from three independent experiments.

Supp_FigS8. Detection of the SulT-Sia activity on CHO cells transfected with *mdkWscd1* and *mdkWscd2* cDNAs. (a) CHO cells are transfected with the plasmid encoding Mock (Mock), mdk*Wscd1* (mdkWscd1) or mdkW*scd2* (mdkWscd2). At 48-h post-transfection, the cells are subjected to FCA with 3G9 (*upper panels*) or IgM control (*lower panels*). (b) The proportions of the 3G9-positive cell population (% area) are shown. All the experiments are triplicate, and the error bars indicate the standard deviations. *p < 0.05 (Student t-test, n=3).

**Supp_FigS9. Generation of *Wscd1*-knockout and *Wscd2*-knockout medaka.** (**a**) *Wscd1*(-/-) strain. Nucleotide sequences for the wild-type and *Wscd1*(-/-) alleles near the target sites. The *Wscd1*(-/-) allele contains 19 bp-deletion in exon 2 (del19). (**b**) *Wscd2*(-/-) strain. Nucleotide sequences for the wild-type and *Wscd2*(-/-) alleles near the target sites. The *Wscd2*(-/-) allele contains 1-bp deletion and 32-bp insertion in exon 1 (in32del1). The target and the protospacer-adjacent motif (PAM) sequences are highlighted by orange and green, respectively.

**Supp_FigS10. Morphological observation of medaka fry at 8 dpf.** WT or *Wscd2*(+/+), *Wscd2*(-/-), and *Wscd1*(-/-) fry are photographed from almost the same direction. The scale bar denotes 200 μm.

**Supp_FigS11.** **Exacerbation of the inflammation state in *Wscd1*(*-/-*) and *Wscd2*(*-/-*) fry at 15 dpf.** The expression level of C-reactive protein (CRP) was determined by qRT-PCR, using β-actin expression was as a control. All the experiments were performed in triplicate. The error bars indicate the standard deviations. *p < 0.05 (Student t-test, n=3).

**Supplementary Figures for the uncropped data for each of the necessary figures**

**Supp_FigS12 for** **Fig_2b.** In this experiment, a PVDF membrane, after electronically blotted, was cut into the upper and lower parts according to the molecular weight, followed by immunostaining with 3G9 for SiaS (upper panel) and with anti-GAPDH for GAPDH (lower panel), respectively. The squared parts are used in Fig. **2**b.

**Supp_FigS13 for Fig_2c.** In this experiment, a PVDF membrane, after electronically blotted, was cut into the left and right parts. The right part was used for chemical analysis. i.e., fluorometric HPLC analysis, whose data is shown in the right panel of Fig. **2**c. The right panel was subjected to immunoblotting with 3G9. The squared part is used in the left panel of Fig. **2**c.

**Supp_FigS14 for Fig_3b and 3e.** **3**b, In this experiment, a PVDF membrane, after electronically blotted, was cut into the upper and lower parts according to the molecular weight, followed by immunostaining with anti-V5 (upper panel) and with anti-GAPDH for GAPDH (lower panel), respectively. The squared parts are used in Fig. **3**b. **3**e, The same experiment was done using a separate PVDF membrane for Fig. 3e. The squared parts are used in Fig. **3**e.

**Supp_FigS15 for Fig_5a.** One TLC plate was used for the analysis of marker, Mock, and Wscd1 (left panel), and the other TLC plate was used for the analysis of Wscd2 (right panel). The three squared parts are put side by side to show Fig. **5**a.

**Supp_FigS16 for Fig_5c.** In this experiment, the same samples were applied to two sets of SDS-PAGE. The one gel was electronically blotted on the PVDF membrane, followed by immunostaining with anti-3G9 (*upper* panel). The other gel, which was lacking in TF only lane corresponding to lane 9 of *upper* panel, was stained with CBB staining (*lower* panel). The squared parts are used in Fig. **5**c.

**Supp_FigS17 for** **Fig_6d.** In this experiment, a large PVDF membrane, after electronically blotted, was cut into the upper and lower parts according to the molecular weight, and the upper parts separated into left and right parts. The lower part was immunostained with anti-β-actin for β-actin. The upper left and right were immunostained with MF20 antibody for myosin heavy chain. The squared parts are used in Fig. **6**d.

**Supp_FigS18 for** **Supp_FigS2a.** Agarose gel electrophoresis profiles of the amplified fragments by the Wscd1- and Wscd2-specific primers using the primary cDNAs from E14.5 mouse embryos. The left squared part is used for the upper panel. The right two squared parts are used for the lower 2 panels (Looks combined).

Supp_FigS19 for Supp_FigS5a and S5d. Agarose gel electrophoresis profiles of the amplified fragments by the Wscd1- and Wscd2- and β-actin-specific primers using the primary cDNAs from HEK and SK-N-SH cells, left 2 panels and right 2 panels, respectively. The squared parts on the left 2 panels and the right 2 panels are used for Supplementary Fig. 5a, and 5d, respectively.

**Table S1. Primer sequences for cloning of mouse and medaka Wscd1 and Wscd2 genes into pGEM-T Easy plasmid**

| Name | Sequence (5’-3’) | Plasmid name |
| --- | --- | --- |
| mWscd1-F  mWscd1-R | ATGGCCAAACCTTTCTTCAGAC  TCATCTGGGCACATACTCTCTG | pGEM-mWscd1 |
| mWscd2-F  mWscd2-R | ATGGCCAAGCTCTGGTTCA  TCATCTTGGGCCATAGGCA | pGEM-mWscd2 |
| mdkWscd1-F  mdkWscd1-R | ATGGGGGTGCCCCTCTACAAG  TCATCTTGTGGGACTGTAATCC | pGEM-mdkWscd1 |
| mdkWscd2-F  mdkWscd2-R | ATGGCCAAGCCTCTCCTGAAGA  TCATCTTGGCATATATTCAGCTGG | pGEM-mdkWscd2 |

**Table S2. Primer sequences for the pcDNA3.1 plasmids**

| Name | Sequence (5’-3’) | Plasmid name |
| --- | --- | --- |
| Wscd1-F  Wscd1-R | AAGCAGTGGTATCAAGCCACCATGGCCAAACCTTTCTTCAG CGGGGTACGATGAGAATCTGGGCACATACTCTCTG | pcDNA-mWscd1 |
| Wscd2-F  Wscd2-R | AAGCAGTGGTATCAAGCCACCATGGCCAAGCTCTGGTTC  GACCGGGGTACGATGAGAATCTTGGGCCATAGGCATCAG | pcDNA-mWscd2 |
| mdkWscd1-F  mdkWscd1-R | GTACCGAGCTCGGATCCGCCACCATGGGGGTGCCCCTCTACAA  TGCTGGATATCTGCAGAATTCTCTTGTGGGACTGTAATCCTCT | pcDNA-mdkWscd1 |
| mdkWscd2-F  mdkWscd2-R | TACCGAGCTCGGATCCGCCACCATGGCCAAGCCTCTCCTGAA  TGCTGGATATCTGCAGAATTCTCTTGGCATATATTCAGCTGG | pcDNA-mdkWscd2 |

**Table S3. Primer sequences for the pcDNA3.1-mutWscd1/mutWscd2 plasmids** Altered nucleotides are shown by the underlined letter.

| Name | Sequence (5’-3’) | Plasmid name |
| --- | --- | --- |
| Wscd1PAPS1-F  Wscd1PAPS1-R | GCTCTGTCGAGCTTCGCTGGAGCTGGGAAC GTTCCCAGCTCCAGCGAAGCTCGACAGAGC | pcDNA-mWscd1-P357A |
| Wscd1PAPS2-F  Wscd1PAPS2-R | AGCTTCGCTGGAGCTGCGAACACATGGGCA TGCCCATGTGTTCGCAGCTCCAGCGAAGCT | pcDNA-mWscd1-P357A/G360A |
| Wscd1PAPS3-F  Wscd1PAPS3-R | GCTGGAGCTGCGAACGCCTGGGCAAGGCAC  GTGCCTTGCCCAGGCGTTCGCAGCTCCAGC | pcDNA-mWscd1-P357A/G360A/T362A |
| Wscd1PAPS4-F  Wscd1PAPS4-R | GGAGCTGCGAACGCCGCGGCAAGGCACCTGA  TCAGGTGCCTTGCCGCGGCGTTCGCAGCTCC | pcDNA-mWscd1-P357A/G360A/T362A/W363A or pcDNA-mutWscd1 |
| Wscd2PAPS1-F  Wscd2PAPS1-R | GCTCTAGCCAGCTTCGCTGGCGCTGGCAAC  GTTGCCAGCGCCAGCGAAGCTGGCTAGAGC | pcDNA-mWscd2-P356A |
| Wscd2PAPS2-F  Wscd2PAPS2-R | AGCTTCGCTGGCGCTGCCAACACGTGGGCT  AGCCCACGTGTTGGCAGCGCCAGCGAAGCT | pcDNA-mWscd2-P356A/G359A |
| Wscd2PAPS3-F  Wscd2PAPS3-R | GGCGCTGCCAACGCCTGGGCTCGCCACCTC  GAGGTGGCGAGCCCAGGCGTTGGCAGCGCC | pcDNA-mWscd2-P356A/G359A/T361A |
| Wscd2PAPS4-F  Wscd2PAPS4-R | GGCGCTGCCAACGCCGCGGCTCGCCACCTCAT  ATGAGGTGGCGAGCCGCGGCGTTGGCAGCGCC | pcDNA-mWscd2-P356A/G359A/T361A/W362A or pcDNA-mutWscd1 |

**Table S4. Oligonucleotides for shRNA plasmids to *Wscd1* and *Wscd2* gene** The target sequences for RNA interference are underlined.

| Name | Sequence (5’-3’) | Plasmid |
| --- | --- | --- |
| shWscd1-F  shWscd1-R | GATCCCCGGAGTTTGTGAATAGCTATTTCAAGAGAATAGCTATTCACAAACTCCTTTTTA  AGCTTAAAAAGGAGTTTGTGAATAGCTATTCTCTTGAAATAGCTATTCACAAACTCCGGG | shWscd1 |
| shWscd2-F  shWscd2-R | GATCCCCGGCTGAGTTCAACCGCAAATTCAAGAGATTTGCGGTTGAACTCAGCCTTTTTA  AGCTTAAAAAGGCTGAGTTCAACCGCAAATCTCTTGAATTTGCGGTTGAACTCAGCCGGG | shWscd2 |

**Table S5. Oligonucleotides for the CRISPR/Cas9 target site-containing pDR274 plasmid** The sequences for CRISPR-Cas9 target site are underlined.

| Name | Sequence (5’-3’) | Plasmid |
| --- | --- | --- |
| sgWSCD1-F  sgWSCD1-R | TAGGAGATGCTGCTGCTCTGCC  AAACGGCAGAGCAGCAGCATCT | pDR274-sgWscd1 |
| sgWSCD2-F  sgWSCD2-R | TAGGATCCAGCGCTACTTCCGC  AAACGCGGAAGTAGCGCTGGAT | pDR274-sgWscd2 |

**Table S6. Primer sequences for qRT-PCRs**

| Name | Sequence (5’-3’) | Target genes and species |
| --- | --- | --- |
| qPCRmWscd1-F  qPCRmWscd1-R | CCTGATACAGGCCAATGTGAC  GGTAGGGTAAGCACAGTAGCA | human *Wscd1* in HEK and SK-N-SH cells |
| qPCRmWscd1-F  qPCRmWscd1-R | GACTACAAAAAGATGACCATC  GTTCGTCGCCTGGATCTTGTG | human *Wscd2* in HEK and SK-N-SH cells |
| qPCRmdkWscd1-F  qPCRmdkWscd1-R | AGGAGAGATTTTCAGCACGC  ACGCATGTCGTACAGCATAG | medaka *Wscd1* in young fry and adult organs |
| qPCRmdkWscd2-F  qPCRmdkWscd2-R | CAGCTGGAATCGGGCACTGA  GCGCTCTTTTCTGAGTGTTG | medaka *Wscd2* in young fry and adult organs |
| qPCRCRP-F  qPCRCRP-R | ATGTGAGGCTGAACCCAACA  CCGTTGTCGTAAGTGGGTGT | medaka *C-reactive protein* in fry |
| qPCRbact-F  qPCRbact-R | TCTTTCCCTCCATCGTTGGTC  TCAGGGTCAGGATACCCCTC | medaka *β-actin* in fry and adult fish |

**Table S7. Primers for genotyping of the knockout allele for Wscd1 and Wscd2**

| Name | Sequence (5’-3’) | Target genes and species |
| --- | --- | --- |
| mdkWscd1-F  mdkWscd1-R | ACAGGCTGGGATATTAAACTGC  CGAAGATCTGAGAGGAAGGTCT | to amplify exon 2 of medaka *Wscd1* |
| mdkWscd2-F  mdkWscd2-R | ACTCACACTTGAGCTTCATGTG  TTCCTCATGTCCACTCTTCCTC | to amplify between upstream and exon 1 of medaka *Wscd2* |

Supp_FigS1. Immunohistochemistry of human tissue sections with 3G9. Sections of adult human tissues obtained from the kidney, liver, brain, breast, skin, and prostate were immunostained with 3G9 and 2G9 (isotype control). The SiaS epitopes were visualized by Alexa-488-conjugated anti-mouse IgM (Alexa, green). Nuclei were stained with DAPI (Dapi, blue).

Supp_FigS2. Amino acid sequences for mWscd1 and mWscd2. (a) RT-PCR of the full-length of *mWscd1* and *mWscd2* cDNAs. The first-strand DNA was prepared from the total RNA extracted from the E14.5 mouse embryonic brain. The *mWscd1* and *mWscd2* cDNAs were amplified from the first-strand DNA using the gene-specific primers (Supplementary Table 1); (b) Amino acid sequence alignments of mouse Wscd1 (mWscd1) and mWscd2. The sequences were deduced from the nucleotide sequences of cDNAs for mouse *Wscd1* and *Wscd2*. They contain two conserved PAPS binding motifs, 5’-PSB and 3’-PB in order (red underline). Accession numbers are Gene ID: 216881 for *Wscd1* and DDBJ LC669910 for *Wscd2*. The mWscd2 cDNA contained three silent mutations (57G>C, 954T>C, and 1125T>C) in Gene ID: 320916;

Supp_Fig2. (continued)

(c) Putative PAPS binding sites in *Wscd1* and *Wscd2*. The *upper panels* display the consensus sequences of two conserved PAPS binding sites, 5’-PSB and 3’-PB, responsible for the binding to the 5’-phosphosulfate group and 3’-phosphate group pf PAPS, respectively, among sulfotransferases [65]. The *lower panel* displays the sequence alignment of mWscd1 and mWscd2 with 5’-PSB and 3’-PB of the mouse estrogen sulfotransferase (mES, marked by *). Red letters in the sequence represent identical amino acid residues of the three.

**Supp_FigS3. Molecular phylogenetic analysis of *Wscd1* and *Wscd2*.** (**a**) Phylogenetic tree of mouse sulfotransferase genes. The tree was generated based on the nucleotide sequences by the neighbor-joining method on Genetyx software. Wscd1 and Wscd2 are highlighted by squares. The Genebank accession numbers are listed in Supplementary Data 1.

**Supp_FigS3.** (continued)

(**b**) Phylogenetic tree of Wscd1 and Wscd2 in deuterostome. The orthologous genes are ubiquitously distributed in deuterostome. *Homo sapiens*, human; *Pan troglodytes*, chimpanzee; *Gorilla gorilla gorilla*, gorilla; *Macaca mulatta*, rhesus monkey; *Mus musculus*, mouse; *Rattus norvegicus*, Norway rat; *Gallus gallus*, chicken; *Anolis carolinensis*, Green anole; *Xenopus laevis*, clawed frog; *Danio rerio*, zebrafish; *Oryzias latipes*, medaka fish; *Branchiostoma floridae*, lancelet; *Ciona intestinalis*, vase tunicate; *Saccoglossus kowalevskii*, acorn worm; *Strongylocentrotus purpuratus*, purple sea urchin. The phylogenetic tree was generated based on the amino acid sequences by the neighbor-joining method usiing Genetyx software. The Genebank accession numbers are listed in Supplementary Data 2.

Supp_FigS4. Structures of mutWscd1 and mutWscd2. The nucleotide and amino acid sequences of the 5’-PSB regions for Wscd1, Wscd2 and their Ala-mutants, mutWscd1 and mutWscd2, respectively.

Supp_FigS5. Effects of knockdown of the Wscd1 and Wscd2 genes. (a) RT-PCR of Wscd1, Wscd2, and β-actin genes in human HEK cells; (b) Expression level of Wscd1 mRNA of HEK cells transfected with shMock or shWscd1 plasmid, as measured by qRT-PCR; (c) FCA of the surface expression of SiaS with 3G9 on HEK cells transfected with shMock or shWscd1 plasmid. (*left* and *middle*) Mock-cells (shMock) and Wscd1-suppressing cells (shWscd1). The proportions of the 3G9-positive cell population (% area) are shown in each panel; (*right*) % Area obtained from the FCA histograms in *left* and *middle* panels. All the experiments were triplicated, and the error bars indicate the standard deviations. *p < 0.05 (Student t-test, n=3). (d) RT-PCR of Wscd1, Wscd2, and β-actin genes in human SK-N-SH cells; (e) Expression level of Wscd1 mRNA of SK-N-SH cells transfected with shMock or shWscd1 plasmid, as measured by qRT-PCR; (f) FCA of the surface expression of SiaS with 3G9 on SK-N-SH cells transfected with shMock or shWscd1 plasmid. See the legend for the *left,* *middle*, and *right* panels. All the experiments were triplicated, and the error bars indicate the standard deviations. *p < 0.05 (Student t-test, n=3).

Supp_FigS6. Deduced amino acid sequences for medaka Wscd1 and Wscd2. The cDNAs for mdk*Wscd1* and mdk*Wscd2* are cloned from the medaka fry at 6 dpf and 7 dpf, respectively. The cDNA sequence for mdkWscd1 (LC669911) contained four silent mutations (701A>G, 1452A>G, 1497T>C, and 1652G>A) and two missense mutations (59T>C, and 311A>G) in Gene ID:101157150, and that for mdkWscd2 (LC669912) contained four silent mutations (564T>C, 1029C>T, 1080T>C, and 1128T>C) and two missense mutations (928T>G, and 1090G>T) in Gene ID:101164728. mdkWscd1 and mdkWscd2 consist of 1695 and 1686 bp encoding 565 and 562 amino acid residues, respectively. These two amino acid sequences show 45 % identities with each other and contain two conserved PAPS binding motifs, 5’-PSB and 3’-PB (red underline).

Supp_FigS7. Expression profiles of *mdkWscd1* and *mdkWscd2* genes. (a) Developmental expression in pre-hatching stage fry. (b) Organ-dependent expression in 3-month-old-adult. The expression levels are quantified by qRT-PCR, and normalized to that of the housekeeping β-actin in the same sample. The error bars represent standard deviations from three independent experiments.

Supp_FigS8. Detection of the SulT-Sia activity on CHO cells transfected with *mdkWscd1* and *mdkWscd2* cDNAs. (a) CHO cells are transfected with the plasmid encoding Mock (Mock), mdk*Wscd1* (mdkWscd1) or mdkW*scd2* (mdkWscd2). At 48-h post-transfection, the cells are subjected to FCA with 3G9 (*upper panels*) or IgM control (*lower panels*). (b) The proportions of the 3G9-positive cell population (% area) are shown. All the experiments are triplicate, and the error bars indicate the standard deviations. *p < 0.05 (Student t-test, n=3).

**Supp_FigS9. Generation of *Wscd1*-knockout and *Wscd2*-knockout medaka.** (**a**) *Wscd1*(-/-) strain. Nucleotide sequences for the wild-type and *Wscd1*(-/-) alleles near the target sites. The *Wscd1*(-/-) allele contains 19 bp-deletion in exon 2 (del19). (**b**) *Wscd2*(-/-) strain. Nucleotide sequences for the wild-type and *Wscd2*(-/-) alleles near the target sites. The *Wscd2*(-/-) allele contains 1-bp deletion and 32-bp insertion in exon 1 (in32del1). The target and the protospacer-adjacent motif (PAM) sequences are highlighted by orange and green, respectively.

**Supp_FigS10. Morphological observation of medaka fry at 8 dpf.** WT or *Wscd2*(+/+), *Wscd2*(-/-), and *Wscd1*(-/-) fry are photographed from almost the same direction. The scale bar denotes 200 μm.

**Supp_FigS11.** **Exacerbation of the inflammation state in *Wscd1*(*-/-*) and *Wscd2*(*-/-*) fry at 15 dpf.** The expression level of C-reactive protein (CRP) was determined by qRT-PCR, using β-actin expression was as a control. All the experiments were performed in triplicate. The error bars indicate the standard deviations. *p < 0.05 (Student t-test, n=3).

**Supplementary Figures** **S12-S19: Uncropped data for each of the necessary figures**

**Supp_FigS12 for** **Fig_2b.** In this experiment, a PVDF membrane, after electronically blotted, was cut as shown in the right panel. PVDF1 was immunostained by 3G9 for SiaS (Upper left panel). PVDF2 was immunostained by anti-GAPDH for GAPDH (Lower middle panel), together with PVDF3, which was obtained from completely different experiment. The squared parts are used in Fig. **2**b. Higher and lower intensified images are also shown (Lower left panels).

**Supp_FigS13 for Fig_2c.** In this experiment, a PVDF membrane, after electronically blotted, was cut into the left and right parts. The right part was used for chemical analysis. i.e., fluorometric HPLC analysis, whose data is shown in the right panel of Fig. **2**c. The right panel was subjected to immunoblotting with 3G9. The squared part is used in the left panel of Fig. **2**c.

**Supp_FigS14 for Fig_3b and 3e.** **3b**, In this experiment, a PVDF membrane, after electronically blotted, was cut into the upper and lower parts according to the molecular weight, followed by immunostaining with anti-V5 (upper panels) and with anti-GAPDH for GAPDH (lower panels), respectively. The squared parts are used in Fig. 3b. A higher exposure image with anti-V5 is shown at the right of the corresponding panel. **3e**, The same experiment was done using a separate PVDF membrane for Fig. 3e. The squared parts are used in Fig. 3e. A lower exposure image with anti-V5 is shown at the right of the corresponding panel.

**Supp_FigS15 for** **Fig_5a.** One TLC plate was used for the analysis of marker, Mock, and Wscd1 (left panel), and the other TLC plate was used for the analysis of Wscd2 (right panel). The three squared parts are put side by side to show Fig. **5**a.

**Supp_FigS16 for Fig_5c.** In this experiment, the same samples were applied to two sets of SDS-PAGE. The one gel was electronically blotted on the PVDF membrane, followed by immunostaining with anti-3G9 (*upper* panel). The other gel, which was lacking in TF only lane corresponding to lane 9 of *upper* panel, was stained with CBB staining (*lower* panel). The squared parts are used in Fig. **5**c. The right two panes show images of different exposures.

**Supp_FigS17 for** **Fig_6d.** **(a)** Three gels were prepared: Gel1 was for Wscd1 samples, Gel2 was for Wscd2 samples, and Gel3 was for Wscd1+Wscd2 samples (only for the house keeping protein detection). Each of them was subjected to SDS-PAGE-Western blotting. The PVDF1 and PVDF2 membranes were used for immunostaining with MF20 antibody for myosin heavy chain. The PVDF3 was immunostained with anti-β-actin for β-actin.

**Supp_FigS17 for** **Fig_6d. (b)** For Wscd1, the PVDF1 was immunostained with MF20 antibody. Three kinds of images of different exposures are shown as image 1, 2, and 3. For image 1 and 3, the images of high contrast are also shown, and clearly indicate the margins. All of them are original and full-length membranes. The squared part in image 1 is used in Fig. **6d**.

**Supp_FigS17 for** **Fig_6d.** **(c)** For Wscd1, the PVDF2 was immunostained with MF20 antibody. Three kinds of image of different exposures, together with their high contrast images, are shown as image 1, 2, and 3 on the left and right panels. The high contrast images clearly show the margins. All of them are original and full-length membranes. The squared part in image 1 is used in Fig. **6d**.

**Supp_FigS17 for** **Fig_6d.** **(d)** For β-actin, the PVDF3 was immunostained with anti-β-actin for β-actin, followed by chemiluminescent detection. Two of them are original and full-length membranes. The squared parts on the top panel are used in Fig. **6d**.

**Supp_FigS18 for** **Supp_FigS2a.** Agarose gel electrophoresis profiles of the amplified fragments by the Wscd1- and Wscd2-specific primers using the primary cDNAs from E14.5 mouse embryos. The left squared part is used for the upper panel. The right two squared parts are used for the lower 2 panels (Looks combined).

Supp_FigS19 for Supp_FigS5a and S5d. Agarose gel electrophoresis profiles of the amplified fragments by the Wscd1- and Wscd2- and β-actin-specific primers using the primary cDNAs from HEK and SK-N-SH cells, left 2 panels and right 2 panels, respectively. The squared parts on the left 2 panels and the right 2 panels are used for Supp_FigS5a, and S5d, respectively.

Supp_DataS1.

Supp_DataS2.

Supp_DataS2. (continued)
